# Bad Teeth and Disease

**Published:** 1882-12

**Authors:** 


### BAD TEETH AND DISEASE.

HAD we the means, we should endow a charity, the great aim of which should be to prevent disease by establishing an institution for the treatment of decayed and imperfect teeth. There are more cases of disease of various kinds and various degrees of severity emanating from bad teeth than from almost any other cause. The trouble is easily remedied if taken in time ; but those who suffer most are they who have not the means to employ competent dentists. It is pitiful to see the children of the poor as they grow up, gradually losing their teeth by decay and neglect, and becoming dyspeptic at twenty, and old and haggard at thirty. If there is a nobler charity than that which would supply free dentistry to the poor, and dentistry at cost to those who are able to pay no more, we know not what it is.

But there are persons of ample means who pay no attention to their own teeth or those of their children. They should be taught the importance of attending to this matter, and if they then refused they should be punished for the neglect of an important duty toward their families. There is no excuse for any person's having bad teeth. A child can be taught the importance of attending to the teeth, and every child that has his second front teeth should be provided with a tooth-brush, and be required to use it at least once every day, using castile soap. Once in six months at furthest a dentist should be employed to examine the teeth and properly fill any that may be found decayed. Were this plan generally adopted, we should see no more toothless men and women.

